# FABRICA: A Bioreactor Platform for Printing, Perfusing, Observing, & Stimulating 3D Tissues

**DOI:** 10.1038/s41598-018-25663-7

**Published:** 2018-05-15

**Authors:** Lester J. Smith, Ping Li, Mark R. Holland, Burcin Ekser

**Affiliations:** 1 Radiology and Imaging Sciences, Indiana University School of Medicine, Indianapolis, IN USA; 2 3D Bioprinting Core, Indiana University-Purdue University Indianapolis, Indianapolis, IN USA; 3 Division of Transplant Surgery, Department of Surgery, Indiana University School of Medicine, Indianapolis, IN USA

## Abstract

We are introducing the FABRICA, a bioprinter-agnostic 3D-printed bioreactor platform designed for 3D-bioprinted tissue construct culture, perfusion, observation, and analysis. The computer-designed FABRICA was 3D-printed with biocompatible material and used for two studies: (1) Flow Profile Study: perfused 5 different media through a synthetic 3D-bioprinted construct and ultrasonically analyzed the flow profile at increasing volumetric flow rates (VFR); (2) Construct Perfusion Study: perfused a 3D-bioprinted tissue construct for a week and compared histologically with a non-perfused control. For the flow profile study, construct VFR increased with increasing pump VFR. Water and other media increased VFR significantly while human and pig blood showed shallow increases. For the construct perfusion study, we confirmed more viable cells in perfused 3D-bioprinted tissue compared to control. The FABRICA can be used to visualize constructs during 3D-bioprinting, incubation, and to control and ultrasonically analyze perfusion, aseptically in real-time, making the FABRICA tunable for different tissues.

## Introduction

Biofabrication/bioprinting is a relatively new and developing field that seeks to produce tissue constructs from cells and either investigate those constructs as models of tissue development or tissue pathology or prepare them as replacements of damaged or destroyed tissues^1–3^. The complex design of most tissues and their need to be perfused with nutrients calls for thoughtful bioreactor design. Specifically, how the tissues will be printed, cultured, perfused, mechanically (or otherwise) stimulated, and analyzed during culture should be carefully considered in bioreactor design^4–7^.

Bioreactor designs and approaches are many but are limited to rudimentary designs and thus rudimentary tissue models which do not simulate the natural tissue perfusion environment^8–14^. Additionally, many of the biofabricated construct designs either are not conducive to perfusion or they do not translate well from bioprinter to bioreactor to implantable use cases^15,16^, meaning that bioreactor developed tissues must be translated into form factors suitable for animal models or clinical use. For example, the perfusion bioreactor platform designs^13,14^ are capable of generating biomimetic macro-vascular networks. However, transferring the biofabricated vascular network to an animal or patient requires destruction of the delicate vascular network. In other examples, perfusion is driven through a porous network whereby the channel diameters and thus flow rates cannot be fully controlled^12^. Without the ability to control flow rates, matching tissue construct perfusion to natural perfusion conditions limits tissue engineers’ ability to control cell differentiation and subsequent tissue formation.

Recent advances of biocompatible, autoclavable, light-cured materials have generated new opportunities for use of these materials in rapid-prototyping (3D printing) complex bioreactor systems. Coupled with scaffold-free biofabrication, biofabricated/bioprinted tissues that can be cultured and transferred from the bioreactor/research setting to advanced use-cases simply and with fewer steps will greatly advance the biofabrication as a clinically useful field.

Another limitation of currently available bioreactors is real-time monitoring^2^. In particular, perfusion by many systems is limited to computational fluid dynamics (CFD)^17^ or the flow is not characterized at all. This means that the actual flow conditions within the construct are rarely understood. Without characterizing the flow conditions within the construct, possible flow losses due to drag effects through the flow channels and possible changes in the flow condition are not accounted for with regard to outcomes and/or tissue viability. Critical flow characteristics include flow velocity within the construct lumen and laminar versus turbulent flow^18,19^. Understanding of the actual flow condition is critical since different tissues have specific flow velocity and flow condition requirements^18,20^ and tissues are sensitive to the fluid shear generated by fluid flow^18,19^.

Additionally, ease-of-use with current bioreactor systems is a design challenge, resulting in difficult construct placement and removal^7^. Difficult handling can lead to operator frustration and error resulting in poorly placed or damaged constructs and high contamination risk.

In this manuscript, the authors describe and introduce the FABRICA, a robust and easy-to-use biocompatible 3D-printed bioreactor capable of containing, culturing, perfusing, and observing biofabricated tissues aseptically and in real-time. Flow through the bioreactor’s integrated channels and into the construct was characterized using ultrasound imaging techniques and the construct was optically observed with a camera through a sensor port. The bioreactor therefore meets 4 of the 5 core principles of a bioreactor system^21^, which are; (i) maintaining sterility, (ii) good mass transfer, (iii) suitability for scale up, and (iv) reproducibility of samples. The fifth, maintaining a controlled metabolic environment, requires tuning the flow rates and imposing appropriate gas mixture into the system is in development, and partly shown in the current manuscript.

## Materials and Methods

### Bioreactor Design

**Figure 1:**
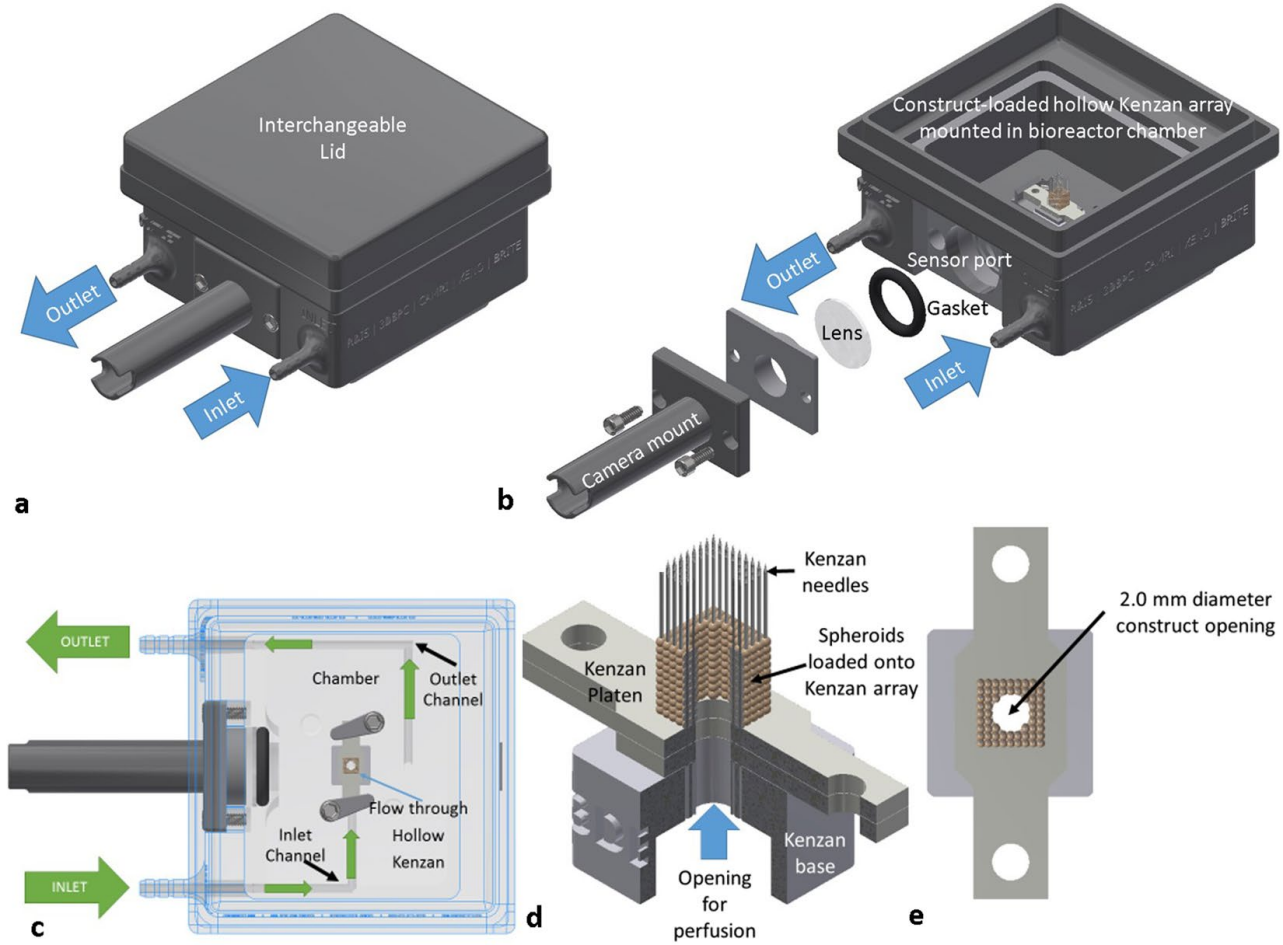
FABRICA Design. (**a**) FABRICA Bioreactor system with the interchangeable lid installed. (**b**) FABRICA bioreactor showing the inlet and outlet ports, how the camera mount is installed onto the camera port, and a tissue construct loaded on Kenzan microneedles within the bioreactor chamber. (**c**) Top-down cutaway showing how constructs are perfused within the FABRICA. (**d**) Representative quarter section rendering of a hollow Kenzan with a perfusible tissue construct loaded onto the needles. (**e**) A top down rendering of the construct on the Kenzan showing the 2 mm diameter opening for perfusive flow.

AutoDesk Inventor Computer-Aided Design (CAD) Software (AutoDesk, San Rafael, CA, USA) was used to design a bioreactor chamber with a front center port for sensor access, and two front ports on either side of the sensor port for fluid inlet and outlet (Fig. 1). The center sensor port can be fitted with interchangeable optical, infrared, or other sensors with modular sensor mounts (Fig. 1b,c). For the present study, a camera mount with a watertight gasket and a lens were installed for optical imaging with a camera (Fig. 1b). The lid opening is low and wide to accommodate easy Kenzan (micro-needle array which is used for a temporary support for scaffold-free 3D bioprinting, Fig. 1d,e) handling and prevent accidental hand/glove contact with the inner bioreactor chamber during construct handling. An interchangeable lid system was also designed so that sensors or actuators could be installed and aseptically interact with the tissue construct within the bioreactor chamber from the top. For this study, a specially-designed proprietary lid for ultrasonic Doppler measurements (not shown) was used. Perfusion channel diameter was 1.5 mm throughout the bioreactor while the construct had a 1.7 mm diameter. The inlet and outlet ports are integrated with 1.5 mm channels that lead to the inner bioreactor chamber (Fig. 1c). The inlet channel is aligned such that the sample holder can contain and direct perfusive flow through the bioprinted construct. The constructs placed onto the FABRICA can be designed with channel openings ranging from 500 μm to 3.0 mm in 500 μm increments (dimensions vary depending on spheroid fusion post-print), accommodating a large range of vascular channel diameters. For the sake of simplicity, not all channel sizes were tested in this study.

### 3D printing of the Bioreactor

**Figure 2:**
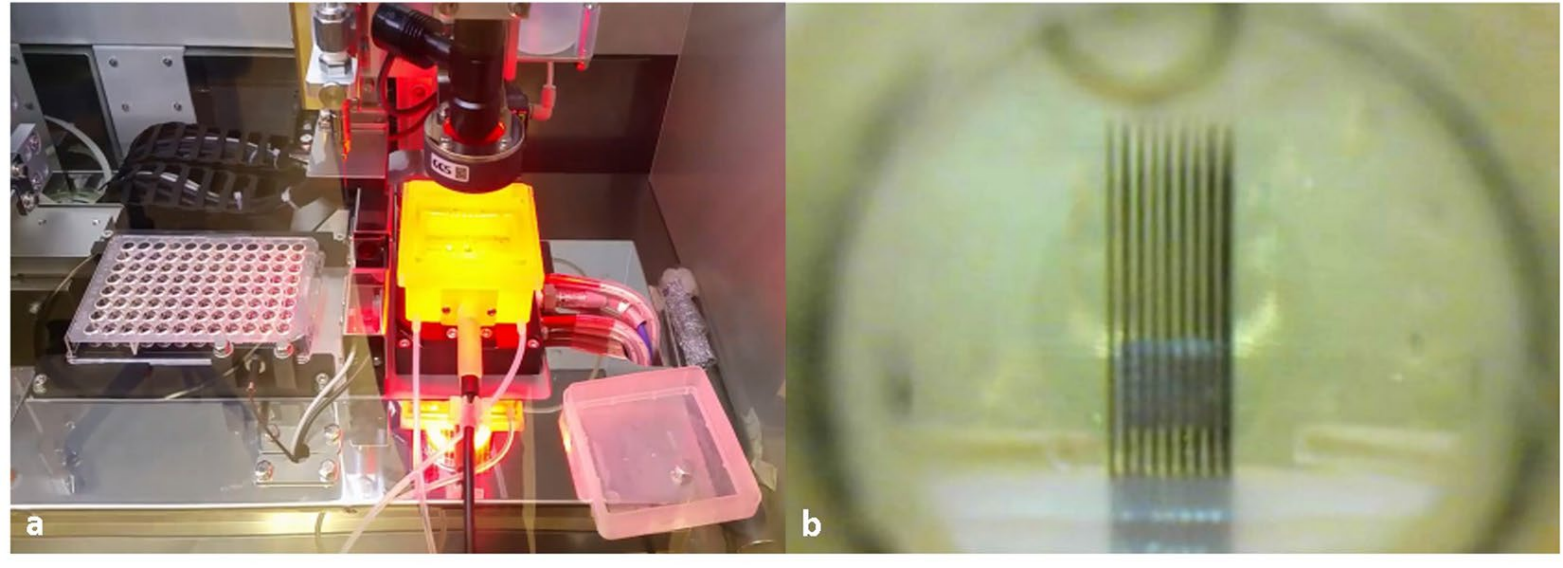
Bioprinting within the FABRICA. (**a**) Image of the FABRICA inside of the Regenova during the bioprint of the tissue construct. (**b**) The camera mounted on the front sensor port of the FABRICA was used to capture images of the construct during printing. The image shown is of the final construct used in the construct perfusion study.

Following CAD design and conversion to an .stl file, PreForm software (Formlabs, Somerville, MA) was used to orient the design for printing, add printing supports to the file design, and then convert the design into a sliced .form file. The .form file was then uploaded to a Formlabs Form 2 stereolithography (SLA) 3D printer and printed using Dental SG, a class I biomaterial dental resin from Formlabs. Once printing was complete, each part was removed from the build plate, washed twice in isopropanol for 10 minutes each, allowed to air dry 4 hours to overnight, and then post-cured in UV light on each side for 20 minutes each. Following post-cure, the parts were assembled, autoclaved, and used (Fig. 2a).

### Optical imaging through sensor port

For optical imaging of the construct used for the perfusion study, a camera mount was installed on the sensor port and a Depstech digital USB endoscope (Depstech, Shenzen, China) connected to an Apple MacBook (Apple, Inc., Cupertino, CA) was installed into the camera mount. Live, real-time, video of spheroid placement onto the construct (bioprinting) was recorded using the video feature on Apple Photo Booth (Apple, Inc., Cupertino, CA) (Fig. 2b). (Supplementary videos 1 and 2).

### Biofabrication and Construct design

The bioprinter is designed for use with 500 µm cell spheroids. For the flow profile experiment, 500 µm agarose beads (which served as a substitute for cell spheroids) (Amuza, Inc., San Diego, CA) were printed on the Kenzan micro-needle array inside of the bioreactor, which was placed inside of a Cyfuse Regenova Bio 3D printer (Amuza, Inc., San Diego, CA, USA). A hollow agarose bead construct 8 beads high (4 mm) with a 3.5 mm outer diameter and a 1.7 mm inner diameter was printed onto the Kenzan array, as previously described^22–24^. Briefly, a computer vision system was used to select a suitable bead and a mobile nozzle with negative pneumatic backpressure was used to pick the selected bead from a 96 well plate. The nozzle then lowers the bead at a predetermined height on a predetermined needle positioned within a 9 × 9 Kenzan needle array. Construct design was achieved using Bio 3D Designer software (Amuza, San Diego, CA). A positive pneumatic back pressure is then applied through the nozzle, releasing the bead which stays in position on the needle array.

Two studies were performed. (i) The first study investigated the flow profiles of several perfusion media with different flow rates (Tables 1 and 2) through a construct composed of nonsterile agarose beads. While the FABRICA in this study is meant to perfuse cellular spheroids that have fused to form a larger construct, the use of a rudimentary construct formed from agarose beads in the first study intended to facilitate proof of concept through demonstration of a flow velocity sweep. While the FABRICA can accomplish the ultrasound flow analysis aseptically on cellular spheroids, the prolonged handling involved in analyzing flow of each fluid at several flow rates would damage a cell-based construct over the course of the experiment. (ii) The second study was a week-long 3D-bioprinted tissue culture and perfusion experiment to investigate the system’s ability to perfuse a scaffold-free 3D-bioprinted construct comprised of cell spheroids and maintain culture conditions within an incubator.

**Table 1:** Medium formulations and flow rates.

Medium Formulations	Pump Flow Rates
Sterile Water + 0.5 mg/mL cornstarch	1 mL/minute
Liver Construct Culture Medium ([LCCM], (αMEM/EGM-MV, 3:1) αMEM (Invitrogen) + EGM-MV (Lonza) + HEPE Buffer + 1% antibiotic + 10% horse serum + 10% FBS + 0.5 mg/mL cornstarch	3 mL/minute
Dulbecco’s Modified Eagle Medium + 0.5 mg/mL cornstarch	5 mL/minute
Porcine Blood (Pig blood + 1 U/mL heparin + 1% antibiotic)	7 mL/minute
Human Blood (Human blood + 1 U/mL heparin + 1% antibiotic)	10 mL/minute

**Table 2:** Medium flow condition at construct center.

Pump Volumetric Flow rate (mL/min)	Water	LCCM	DMEM	Pig Blood	Human Blood
1	nc	nc	nc	nc	nc
3	L	nc	nc	T	T
5	L	L	L	T	T
7	L	L	L	T	T
10	L	L	L	T	T

Legend: nc = not conclusive, T = Turbulent flow, L = Laminar flow.

For the construct perfusion study, a combination of porcine fibroblasts and genetically-engineered liver-derived cells which were CD31^+^ (40,000 cells/well) was seeded into low-binding u-bottom 96-well plates and cultured in culture medium for 72 hours. Unable to adhere to the well, the cells aggregate, forming cell spheroids approximately 500 µm in diameter. The Regenova was then used to print a hollow 5-spheroid-high tissue construct with a 3 mm outer diameter and a 2 mm inner diameter.

### Perfusion and culture

**Figure 3:**
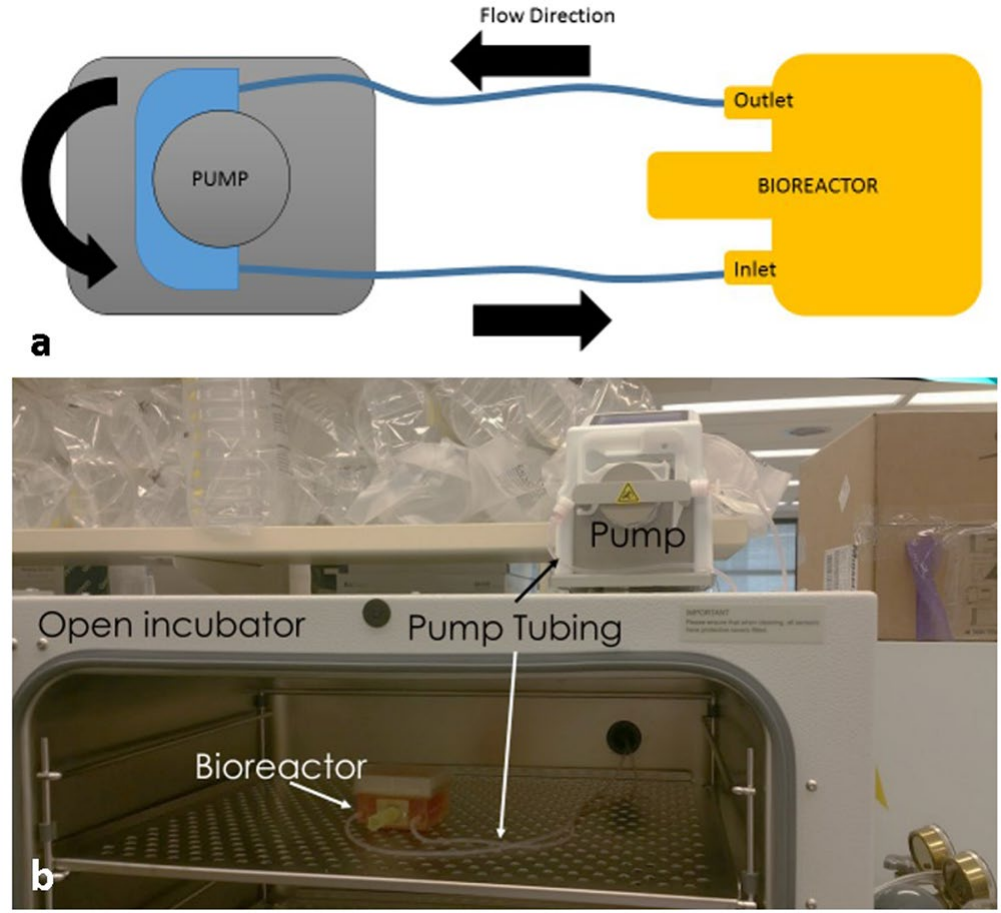
FABRICA perfusion and incubation. (**a**) Schematic of the bioreactor-pump setup showing. (**b**) The FABRICA bioreactor is placed into the incubator and tubing ported through the rear of the incubator is connected to a peristaltic pump.

Silicone tubing (1.52 mm inner diameter, Cole-Parmer Ismatec, Wertheim, Germany) was secured to the inlet and outlet ports, thus producing a closed-circuit flow loop (Fig. 3a). Medium was then poured into the bioreactor and the tubing connected to a peristaltic roller pump. For the flow profile study, each medium (Table 1) was then separately perfused through the bioreactor at each of the volumetric flow rates as listed in Table 1 using a peristaltic pump (REGLO, Cole-Parmer Ismatec, Wertheim, Germany). Cornstarch (0.5 mg/mL)^25^ was included to provide a reflective medium necessary for producing measurable ultrasound signals in water, and 2 other culture media, as indicated in Table 1. Heparinized water was used to flush the bioreactor and tubing clean between measurements made for each of the individual media. For the perfusion study, the tubing was connected to the bioreactor prior to autoclaving and the media was poured into the bioreactor aseptically prior to printing the construct so that the closed flow loop isolated the culture from potential contaminants once the lid was installed. The 3D-bioprinted construct was continuously perfused with 50 mL of culture medium at a rate of 10 mL/minute for one week in the incubator with one medium exchange after 3 days (Fig. 3b).

### Ultrasonic measurements using Doppler ultrasound

**Figure 4:**
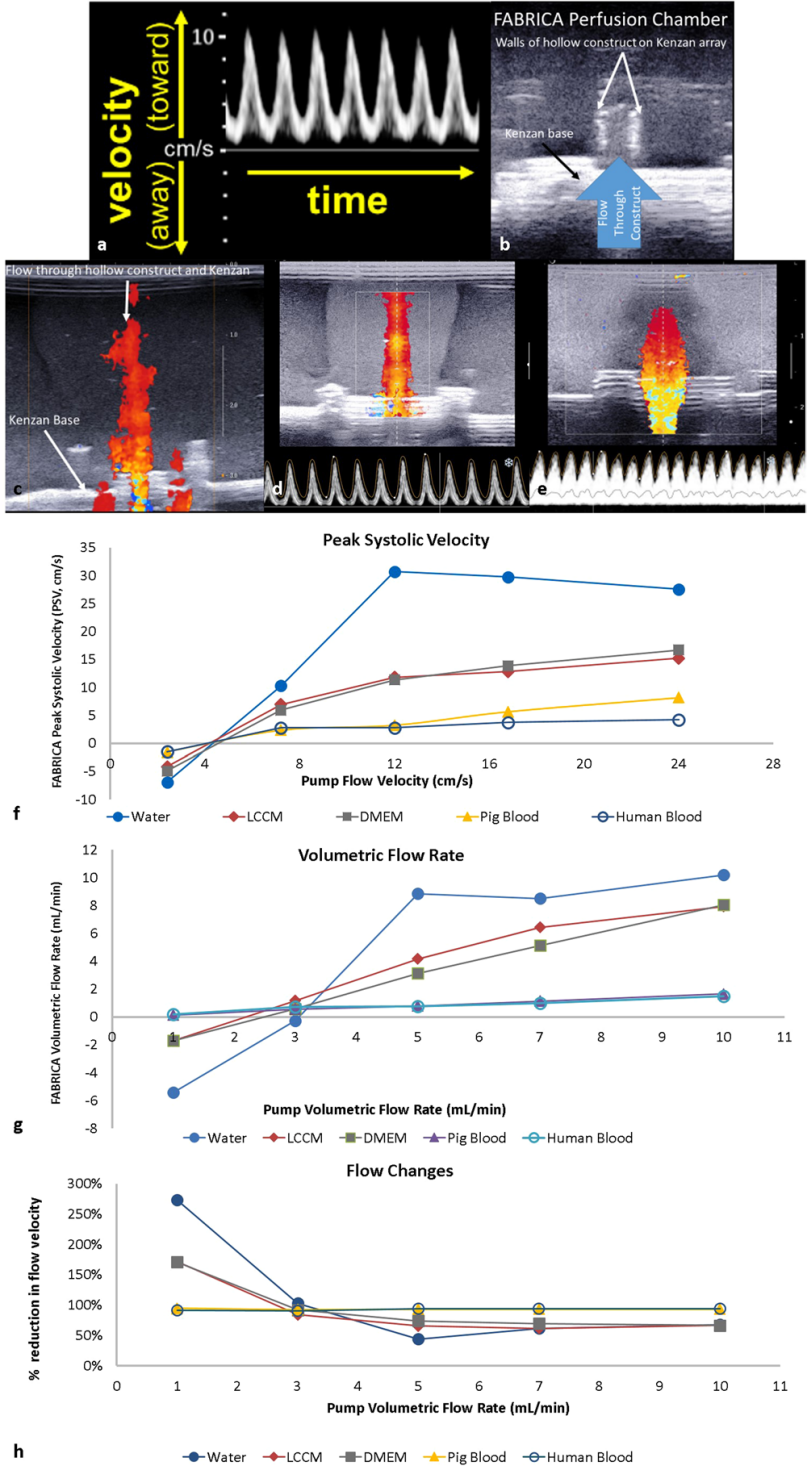
Ultrasonic imaging of construct and flow within the FABRICA. (**a**) Spectral Doppler Waveforms. (**b**) Ultrasonic images of a construct on a Kenzan within the bioreactor where walls of hollow construct were visible. (**c**) Color Doppler image of flow through a hollow construct loaded onto a Kenzan needle array. (**d**) Representative Aixplorer output of the Color Doppler image and Pulse-Wave Spectrum of flow through the construct within the bioreactor with water at 7 mL/minute. The clear waveform with no signal beneath the waveline indicates a narrow velocity spectrum, denoting that flow within the power Doppler gate is laminar. (**e**) Perfusion of pig blood at 10 mL/minute. The waveform shows signal beneath the waveline, indicating a broad velocity spectrum indicative of turbulent flow. The scale on the right of both images is in centimeters. (**f**) Measurements of volumetric flow rate (VFR) within a 3D-bioprinted construct within the FABRICA bioreactor platform using 3 different perfusion media (water, LCCM, DMEM), and porcine and human blood. (**g**) Measurements of time averaged mean velocity (TAMV) within a 3D-bioprinted construct within the FABRICA bioreactor platform using 3 different perfusion media (water, LCCM, DMEM), and porcine and human blood. (**h**) Percentage flow velocity losses through the FABRICA system from the pump to the construct.

To assess the flow characteristics through the hollow agarose bead construct, Color Doppler cineloops and Pulse-Wave Spectral Doppler images were acquired using a commercial ultrasound imaging system (Aixplorer, SuperSonic Imagine, Aix-en-Provence, France) with a high-frequency SL22-7Lab linear transducer array (nominal bandwidth from 7 to 22 MHz). The transducer was placed onto the specially designed lid covering the construct and coupled using a small amount of water within the well surrounding the acoustic window of the specially-designed lid. The Doppler images were obtained with the primary flow direction parallel to the insonifying beam direction. The Doppler gain, velocity scales, baseline, and wall filter settings were optimized to provide robust measurements. Both of the Doppler imaging modes were used to ascertain the flow condition (laminar or turbulent) and flow velocities through the fluid-construct. Flow characteristics were measured by placing the Pulse-Wave Doppler acquisition gate at the center of the lumen between the construct wall boundaries to record the velocity spectrum (Fig. 4a). Subsequent to pulse-wave Doppler data acquisition, the flow characteristics through the construct for each of the media used and flow rates applied were quantified using the automated pulse-wave Doppler measurement tools provided by the Aixplorer ultrasound imaging system. Measurements of the peak and mean velocities were obtained. The corresponding flow rates through the construct were determined using the measured mean velocity values and the known cross-sectional area of the lumen.

### Histology Methods

Scaffold-free 3D-bioprinted porcine liver tissues were either kept in culture medium or continuously perfused using the FABRICA for one week. At the end of the experiment, liver tissue constructs were fixed in 10% formalin for histology. Embedded in paraffin, 4 μm thick sections of the constructs were cut and stained with hematoxylin and eosin (H&E). Histology images were acquired using a Leica DM3000 microscope and Leica Application Software 4.1 (Leica, Wetzlar, Germany).

## Results

The FABRICA, a robust and easy-to-use biocompatible 3D-printed bioreactor capable of containing, culturing, perfusing, and observing biofabricated tissues aseptically and in real-time was successfully designed, produced, and used for (i) a flow profile study with Doppler ultrasound and (ii) a 3D-bioprinted construct perfusion study (Figs 1–3).

The hollow construct of the Kenzan micro-needle array (Fig. 1d), the Kenzan base (Fig. 1d), and the flow through hollow construct inside of the bioreactor could easily be visualized using Doppler ultrasound (Fig. 4b,c). All media (Table 1) exhibited a column of medium flowing through the construct and a mushroom shaped plume at the top of the column which circulated into the chamber (Fig. 4d). Some color Doppler images exhibited additional smaller columns alongside the center column (Fig. 4c,e), indicating flow leakage where the Kenzan is secured into the bioreactor, which may contribute to reductions in VFR and time averaged maximum velocity (TAMV) at the construct.

### Flow profile study

The VFR increased monotonically in positive correspondence with the flow rate applied by the pump. Water showed a sharp rise and then plateaued at 5 mL/minute. Liver Cell Culture Medium (LCCM) and Dulbecco’s modified eagle medium (DMEM) each showed linear increases in volumetric flow rate while the pig and human blood showed shallow increases in VFR. The relation of VFR at the construct (cVFR) or the VFR imposed by the pump (pVFR) varied by medium type, suggesting significant influences to viscosity and flow rate (Fig. 4f).

The TAMV of each medium also increased in correspondence with the flow velocity imposed by the peristaltic pump (Fig. 4g). Low viscosity water showed a linear increase in TAMV while the viscosity of LCCM and DMEM leveled-off of at higher peak systolic velocity (PSV) values (Fig. 4g). High viscosity pig and human blood both showed shallow increases in TAMV (Fig. 4g). Flow velocity changes between the pump and the construct were generally flat for all media beyond 3 ml/min (Fig. 4h). Non-blood media showed a reduction of flow velocity between 44% to 74%. Changes were greater in more viscous blood media, ranging from 90% to 95% between the lowest and highest flow velocities, respectively (Fig. 4h).

### Flow Condition

Water showed clear spectra for 3–10 ml/min pVFR and LCCM, and DMEM showed clear spectra wavelines for 5–10 ml/minute pVFR, indicating laminar flow at these flow parameters. Blood (pig and human) showed broad velocity spectra for all flow rates, suggesting turbulent flow (Table 2).

The volumetric flow rate, flow velocity, and flow condition data for 1–3 ml/minute flow rates were not completely conclusive due to reduced probe sensitivity to low flow conditions (Table 2).

### Construct Perfusion study

**Figure 5:**
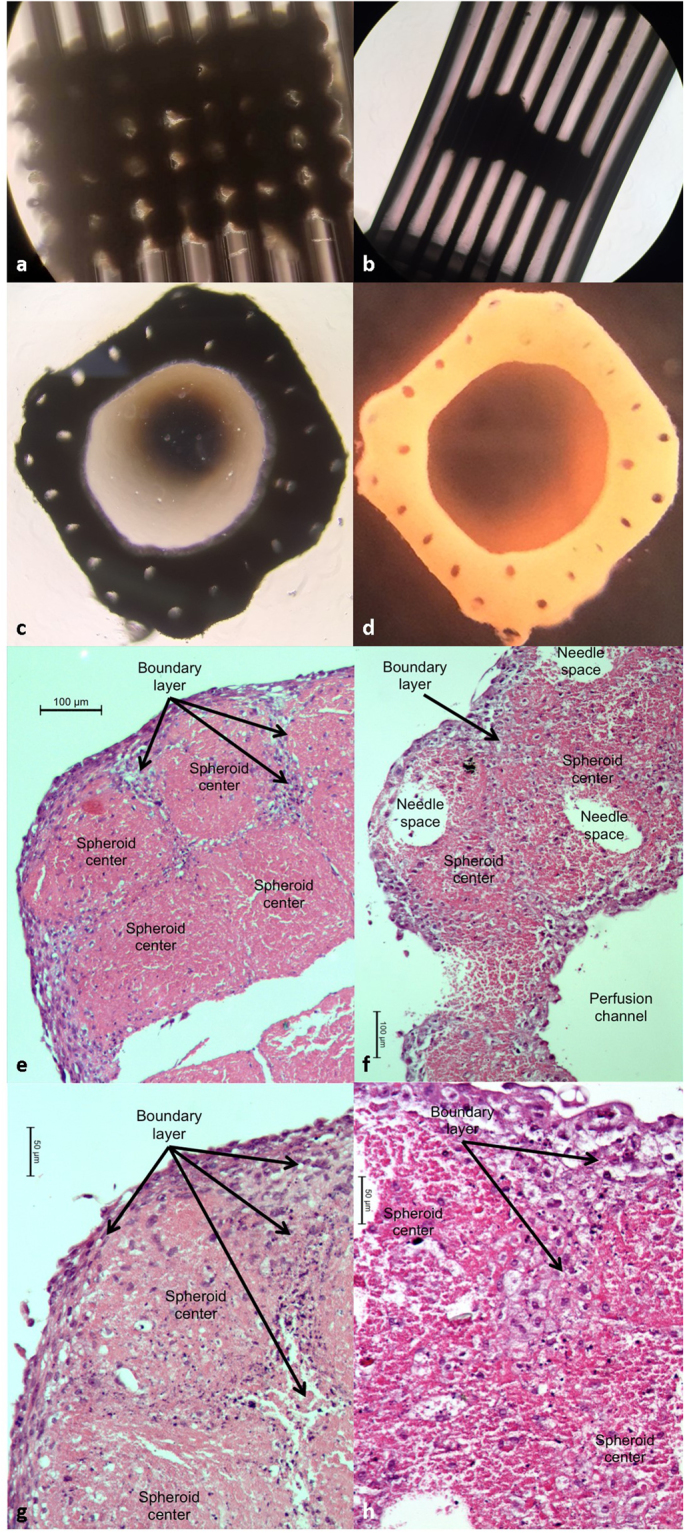
3D-bioprinted construct. (**a**) Microscope image of the 3D-bioprinted tissue construct immediately after printing on day 1. (**b**) Microscope image of the 3D-bioprinted tissue construct on the Kenzan micro-needles after a week of continuous pulsatile perfusion. (**c**) Bright field image of the same construct after its removal from the Kenzan. (**d**) Photograph of the perfused 3D-bioprinted construct after a week of continuous pulsatile perfusion. (**e**) Histology of the scaffold-free 3D-bioprinted liver tissue which was kept statically in the culture media for one week. Solid bar indicates 100 µm. (**f**) Histology of the scaffold-free 3D-bioprinted liver tissue which was continuously perfused using the FABRICA for one week, showing improved survival of cells in the tissue. Solid bar indicates 100 µm. (**g**) Histology of the scaffold-free 3D-bioprinted liver tissue which was kept statically in the culture media for one week. Solid bar indicates 50 µm. (**h**) Histology of the scaffold-free 3D-bioprinted liver tissue which was continuously perfused using the FABRICA for one week, showing improved survival of cells in the tissue. Solid bar indicates 50 µm.

A complete tissue construct was 3D bioprinted with individual spheroids contacting one another on day 1 (Fig. 5a). The LCCM medium was continuously perfused for a week using the FABRICA bioreactor with no visible indicators of contamination or leakage inside the incubator (Fig. 3). The construct contracted along its long axis to about 800 μm in height and the sides were approximately 2.4 mm by 2.4 mm in both length and width and the spheroids fused, resulting in a complete tissue construct (Fig. 5b). The 3D-bioprinted construct was completely patent when removed from the Kenzan microneedles and the luminal opening within the construct center was smooth (Fig. 5c,d). Small holes in the 3D-bioprinted construct where the Kenzan microneedles penetrated were visible with the naked eye after removal (Fig. 5d) (23). Histology of 3D-bioprinted constructs (kept in media statically without perfusion, and perfused with media for one week) was compared. It was shown that there were more viable cells in the perfused 3D construct compared to one kept in culture media statically (Fig. 5e,f). Constructs cultured in static medium demonstrated a thick wall of cell nuclei around the outside of the construct while the spheroid centers showed few or no visible nuclei (Fig. 5e,g). Boundaries, formed by walls of cell nuclei around the spheroids and within the constructs, were visible in the static constructs, suggesting that limited nutrient access to within the spheroids lead to cell death and the interspheroidal spaces between the spheroids was limited to a degree that cell-cell fusion between spheroids could not be accomplished. For the perfused constructs, there were more visible cell nuclei in the core of the spheroid regions while boundaries between the spheroids were less apparent (Fig. 5f,h), though visible, suggesting that perfusion contributed to cell survival within the spheroid centers and the bulk of the construct which was facilitating fusion between spheroids.

## Discussion

As tissue engineering techniques and tissue models continue to advance^1,3,22,26–29^, it is critically important to develop effective methods for the long-term culture, condition, and observation of tissue engineered constructs^5,7,8,12,17,21,26^. Therefore, advanced bioreactors are needed to advance biofabrication, bioprinting, and tissue engineering. We have developed and demonstrated an advanced customizable bioreactor platform capable of simulating the *in vitro* culture environment while providing real-time observation capabilities and ensuring ease-of-use throughout the biofabrication workflow. This bioreactor design allows the real-time observation of tissue construct printing, application of controllable perfusive flow, and real-time ultrasonic observation of perfusion flow, all aseptically.

Different cell types and tissue engineering experiments require different culture media in order to study the desired pathology and obtain desired outcome. However, physiological need for any given cell line, or 3D-bioprinted tissue would be blood which will provide necessary nutrients and oxygen. In the present study, although blood (pig and human) flow showed broad velocity spectra for all flow rates which suggests turbulent flow, the flow parameters imposed on the blood media may not have been sufficient to generate fully conditioned flow for each 3D construct. The range of physiological flow rates should be carefully considered for the given 3D-bioprinted tissue (liver, bone, skin, etc.) and its size. For example, the flow rate imposed in this study (10 mL/minute for the 3D-bioprinted construct for one week) was well beyond the flow rate needed for the size of the 3D-bioprinted tissue.

Doppler ultrasound is widely used in the clinic to characterize flow parameters for each organ and tissue. In tissue bioengineering, there are several examples where Doppler ultrasound was introduced in research experiments to analyze flow parameters for each tissue model^30–32^. In the current study, we were able to characterize the flow velocity, volumetric flow rate, and the flow condition at the construct and the resultant shear stress that develops at the flow boundary. Importantly, tissues are sensitive to each of these parameters and must be specified for each tissue type. For example, peripheral arterial vessel diameters can reach 4 mm^33,34^ and blood flow velocities can reach 50+ cm/second^35^, which is in the range of the flow velocity observed in this study. At the lower dimensional scale, we can generate construct channels diameters as low as 170 µm in diameter spaced 400 µm apart (unpublished), which is on the scale of highly perfused tissues such as liver and bone. The bone has tissue subunits (osteons) with 25–150 µm diameter channels (Haversian canals) spaced 50–400 µm apart^36^ and microvasculature spacing within liver subunits (lobules) is on the order of 500 µm^20^. Therefore, our ability to control the flow parameters and determine actual flow conditions at the construct aseptically in real-time means that the FABRICA bioreactor system can provide the flow profile necessary for a particular tissue model. Furthermore, the bioreactor can be used to meet our long-term goal of conditioning tissue constructs and controlling tissue formation by tuning bioreactors to generate 3D tissue culture profiles (flow, mechanical, or otherwise) matching the physiological profiles of the tissues being engineered. Critically, the ultrasonic analysis of flow and other observations within the FABRICA can be performed aseptically while the tissue is being incubated, allowing changes in flow or gross construct morphology to be monitored and adjusted over the course of a study.

The limitation of the current FABRICA design for the bioreactor system could be related to the studied tissue type (liver, bone, skin, etc) and medium (culture medium, or blood [human or animal]). Therefore, the bioreactor design should be optimized for the type of medium used by adjusting the channel diameter to accommodate higher or lower volumetric flow rates and pumps to impose the needed (higher/lower) volumetric flow rates. Rapid prototyping will help advance the bioreactor design and its implementation very quickly. Rapid prototyping (changing channel diameters and modifying other FABRICA dimensions) is simple since the design can be updated in the CAD file. An updated FABRICA can be 3D-printed in a matter of hours. The modular design of the system means that a different pump can be easily connected to the newly 3D-printed bioreactor. Thus, the FARBICA flow profile can be ultrasonically characterized and a tissue construct can be printed/incubated in the bioreactor all in a matter of days, significantly advancing the pace of tissue engineering research. It should be noted that constructs printed onto the FABRICA can be designed with channel openings ranging roughly from 500 μm to 3.0 mm in 500 μm increments (exact dimensions vary depending on spheroid fusion post-print), accommodating a large range of vascular channel diameters. For proof of concept, only one channel diameter was analyzed in this study.

The compact bioreactor design also means that the 3D construct can be printed directly inside of the bioreactor, limiting the need to handle construct after biofabrication and further reducing both contamination risk and risk of damage to the delicate construct. The reduction of handing steps permitted by the use of integrated perfusion and sensor ports, modular design, and direct bioprinter-to-incubator workflow also serves to reduce fatigue and frustration that comes from difficult installation, which can lead to user error, mis-loaded/printed constructs, and possible contamination. Bioreactor sterility is maintained by having a closed-circuit system which can be autoclaved as a unit and then perfused with medium/blood without having to expose culture medium/blood to pumping components or other potential contaminant sources. The modular design of the FABRICA allows the installation of further sensors and access ports onto the bioreactor so that more advance stimulation (cyclic mechanical loading, electrodes, etc.), culture conditions (i.e., oxygen tension), and sensors (pH, temperature, glucose, stress, strain, etc.) can be applied to the system, advancing the types of tissue models and complexity of data that can be garnered from those models. Therefore, considerate design (which is easy to implement) will improve bioreactor utility and tissue engineering outcomes.

The FABRICA bioreactor is also designed to be bioprinting platform and tissue construct-agnostic, meaning that, with a few minor adjustments, the bioreactor has the potential to culture and condition tissues of any design and constructs generated by scaffold-free and scaffold-dependent methods implemented by the Cyfuse Regenova, the RegenHu Discovery^37^, The EnvisionTec 3D Bioplotter^38^, other biofabrication systems^1^, and otherwise.

Although spheroids may not serve as a suitable model for all tissues and organs, the use of spheroids as building blocks of larger tissue constructs has merit, particularly with regard to the high cell content of spheroid-based tissue constructs (scaffold-free) compared to constructs generated by mixing cells with a biomaterial scaffold (scaffold-dependent) such as collagen or matrigel. Scaffolds interfere with cell-cell communication, which is critical to tissue development at both local structural levels and at organ and limb patterning levels. Spheroids are optimized for perfusion since the diffusion limit in many tissues is on the order of 200–250 μm^39^, which means the core of our spheroids will still receive nutrition. To date, we have accomplished bioprints using spheroids made from bone, muscle, and liver cells and each have shown viability ranging from 1 to 3 weeks. The next level of sophistication, which can be accomplished using current or new biofabrication methods, is to replicate the form of each tissue type with respect to perfusion channel (vascular channel) spacing and the types of cells added to the spheroids/construct. There are some tissues for which the Kenzan method may not be suitable, however, the construct design and biofabrication method-agnostic nature of the FABRICA (which is the focus of this paper) coupled with its Rapid prototyped production method provides the flexibility needed to optimize the FABRICA.

Advances in scaffold-free 3D-bioprinting of genetically-engineered porcine cells^24^, and the FABRICA bioreactor system, will also serve in advancing the field of xenotransplantation^40,41^, transplant immunology, and organ and tissue preservation techniques^42^. For example, organ-specific scaffold-free 3D-bioprinting of genetically-engineered porcine liver^24^ and its continuous perfusion with human blood may give the specific immunological and coagulative response without the need for generating an entire genetically-engineered pig. This reduces the time and budget significantly for each genetic combination, which could cost in excess of $100,000 with the available 28 human and porcine genes available to xenotransplantation research^40–42^. Another example of future advancement with the FABRICA is the perfusion of human blood through the scaffold-free 3D-bioprinted human tissue (liver, kidney, bone, etc.) for the study of blood type ABO incompatible hurdles in an organ specific manner, and the effect of immunosuppressive medications at the given rate and tissue type^43^.

Ongoing studies are centered around computationally defining the flow conditions in more complex construct designs, optimizing the flow to tissue specific perfusion profiles, and developing stimulation approaches, culture conditions, and sensing modules for the system on a Cyfuse Regenova 3D bioprinter. In addition, long-term studies (>3 months) are needed to assess the influence of perfusion on tissue development using this system are in progress.

## Electronic supplementary material

FABRICA Camera Port Video

Regenova + FABRICA
